# Update on Non-Interchangeability of Botulinum Neurotoxin Products

**DOI:** 10.3390/toxins16060266

**Published:** 2024-06-10

**Authors:** Mitchell F. Brin, Mariana Nelson, Nazanin Ashourian, Amy Brideau-Andersen, John Maltman

**Affiliations:** 1 AbbVie/Allergan Aesthetics, Irvine, CA 92612, USA; amy.brideauandersen@abbvie.com (A.B.-A.); john.maltman@abbvie.com (J.M.); 2 Department of Neurology, University of California, Irvine, CA 92697, USA; 3 AbbVie, Irvine, CA 92612, USA; mariana.nelson@abbvie.com; 4 HireGenics, Duluth, GA 30096, USA; nazanin.ashourian@abbvie.com

**Keywords:** botulinum, progenitor toxin complex, SNAP-25, manufacturing, potency, reference standard, duration, immunogenicity

## Abstract

The growing use of botulinum neurotoxins (BoNTs) for medical and aesthetic purposes has led to the development and marketing of an increasing number of BoNT products. Given that BoNTs are biological medications, their characteristics are heavily influenced by their manufacturing methods, leading to unique products with distinct clinical characteristics. The manufacturing and formulation processes for each BoNT are proprietary, including the potency determination of reference standards and other features of the assays used to measure unit potency. As a result of these differences, units of BoNT products are not interchangeable or convertible using dose ratios. The intrinsic, product-level differences among BoNTs are compounded by differences in the injected tissues, which are innervated by different nerve fiber types (e.g., motor, sensory, and/or autonomic nerves) and require unique dosing and injection sites that are particularly evident when treating complex therapeutic and aesthetic conditions. It is also difficult to compare across studies due to inherent differences in patient populations and trial methods, necessitating attention to study details underlying each outcome reported. Ultimately, each BoNT possesses a unique clinical profile for which unit doses and injection paradigms must be determined individually for each indication. This practice will help minimize unexpected adverse events and maximize efficacy, duration, and patient satisfaction. With this approach, BoNT is poised to continue as a unique tool for achieving individual goals for an increasing number of medical and aesthetic indications.

## 1. Introduction

Botulinum neurotoxins (BoNTs) are locally injectable biological medications that are used to treat medical and aesthetic indications. Over the last four decades, clinical use has expanded from the treatment of strabismus and blepharospasm with onabotulinumtoxinA (onabotA) [1,2] to a variety of other neurologic, urologic, dermatologic, and aesthetic indications [3,4]. The growing popularity of BoNT injections has led to a marked increase in the number of available products since the original approval of onabotA in 1989 (Table 1).

As additional BoNT products enter the market, it is increasingly important for clinicians to be aware of differences in formulations, doses, serotypes, and immunogenicity that can impact safety and efficacy [5]. The intrinsic differences among BoNTs impart unique physiochemical characteristics that result in distinct interactions with the tissue microenvironments into which they are injected. Additionally, units of BoNT products are not interchangeable due to differences in the assays used to measure unit potency, including methods for determination of potency reference standards. The non-interchangeability of units among BoNT products led the United States Food and Drug Administration (FDA) to adopt unique, established nonproprietary names for each BoNT product (Table 1). In 2000, with the introduction of a serotype B product, the US FDA began requiring a statement in the prescribing information of each product indicating that units are not interchangeable or convertible among BoNTs. Our initial review published in 2014 examined some of the reasons for non-interchangeability of BoNTs [5]. In view of additional data, coupled with new products on market and in development, the current update seeks to clarify and expand on the basis of non-interchangeability of BoNTs within this new milieu.

**Table 1:** BoNT products commercially available or in development in selected regions worldwide.

Trade Name(s)	Nonproprietary USAN Name	Manufacturer	Serotype	Complex Size or NT Only	Formulation	Selected Regions Approved *
Commercially available						
BOTOX^®^, BOTOX^®^ Cosmetic, Vistabel^®^, Vistabex^®^ [4,6]	OnabotulinumtoxinA	Allergan/AbbVie	A	~900 kDa	In 100 U vial 900 μg sodium chloride 500 μg human serum albumin Finishing: vacuum dried	USA, Canada, EU, China, Japan, South Korea, Brazil
Dysport^®^, Azzalure^®^ [7,8]	AbobotulinumtoxinA	Ipsen	A	~400 kDa **	In 500 U vial 2.5 mg lactose 125 μg human serum albumin Finishing: lyophilized	USA, Canada, EU, China, South Korea, Brazil
Xeomin^®^, Boucouture^®^ [9]	IncobotulinumtoxinA	Merz	A	~150 kDa	In 100 U vial 4.7 mg sucrose 1 mg human serum albumin Finishing: Lyophilized	USA, Canada, EU, Japan, South Korea, Brazil
Nabota^®^, Jeuveau^®^, Nuceiva^®^ [10,11]	PrabotulinumtoxinA	Evolus/Daewoong	A	~900 kDa	In 100 U vial 900 μg sodium chloride 500 μg human serum albumin Finishing: vacuum dried	USA, Canada, EU, South Korea, Brazil
Daxxify™ [12]	DaxibotulinumtoxinA-lanm	Revance	A	~150 kDa	In 100 U vial 0.14 mg L-histidine 0.65 mg L-histidine-HCl monohydrate 0.1 mg polysorbate 20 11.7 µg RTP004 peptide 36 mg trehalose dihydrate Finishing: lyophilized	USA
Myobloc^®^ [13,14]	RimabotulinumtoxinB	Solstice	B	~700 kDa	In 5000 U vial 5.8 mg sodium chloride 470 μg human serum albumin 2.7 mg sodium succinate Finishing: liquid	USA, Canada
Alluzience^TM^ (EU) [8,15]	AbobotulinumtoxinA solution for injection	Ipsen	A	~400 kDa	1.55 mg L-histidine 4.0 mg sucrose 8.76 mg sodium chloride 0.10 mg polysorbate-80 0.10 mg, hydrochloric acid to Finishing: liquid (in water for injection	EU
Neuronox^®^/Meditoxin^®^	Unassigned	Medytox	A	NR	Information from the manufacturer could not be identified.	South Korea, Brazil
Innotox^®^	Unassigned	Medytox	A	NR	Information from the manufacturer could not be identified.	South Korea (approved in 2018; product not available at the time of manuscript submission)
Botulax^®^ (Korea) [16], Letybo^®^ [17] EU: 50 U vial only	LetibotulinumtoxinA	Hugel	A	NR	In 100 U vial 0.9 mg sodium chloride 0.5 mg HSA Finishing: lyophilized	Canada, EU, China, South Korea, USA
Relatox^®^ [18]	None established	Microgen	A	NR	In 100 U vial: 6 mg gelatin 12 mg maltose Finishing: lyophilized	Russia
Hutox^®^ (Liztox^®^) [19]	None established	Huons	A	900 kDa	NR	South Korea
Lantox^®^ (Hengli^®^, Prosigne^®^, Lantox^®^, Lazox^®^ Redux^®^, Liftox^®^) [20,21,22]	None established	Lanzhou	A	900 kDa	In 100 U vial: 5.0 mg gelatin 25 mg dextran 25 mg sucrose Finishing: lyophilized	EU, China, South Korea, Brazil
In Development						
NR [23,24]	RelabotulinumtoxinA	Galderma	A	~150 kDa	Saline phosphate buffer (salt amounts not reported) Finishing: liquid	
NR [25,26]	TrenibotulinumtoxinE	Allergan Aesthetics, an AbbVie company	E	NR	NR	

HCl = hydrochloric acid; HSA = human serum albumin; kDa = kilodalton; mg = milligram; NR = not reported; NT = neurotoxin; U = unit; USAN = United States Adopted Name; μg = microgram. * Approved for one or more indications in the listed countries/regions as of August 2023 based on a search of publicly available information and, for Korean approvals, Wee and Park, 2022 [27]. Specificity of indications and trade names vary from country to country based on local regulatory approvals. See local prescribing information for current indication specifics, including any limitations of use, warnings and precautions, dosage and administration, and adverse reactions. ** The molecular size of the abobotulinumtoxinA neurotoxin complex has been reported to be heterogeneous [28].

## 2. Properties of Botulinum Neurotoxins

### 2.1. Structure

BoNTs are large, multi-domain proteins synthesized by various strains of *Clostridium botulinum* bacteria and are among the most potent substances known, active in the nanogram range [29]. BoNTs are produced as progenitor toxin complexes (PTCs) consisting of a ~150 kDa neurotoxin protein component in association with different-sized naturally occurring neurotoxin associated proteins (NAPs). Each strain produces a non-toxin, non-hemagglutinin (NTNH) protein, that binds in a handshake-like configuration to BoNT, which stabilizes both proteins against low pH and proteases [30,31,32]. Some strains also produce non-toxin hemagglutinin (HA) or other proteins that associate with the neurotoxin/NTNH to form larger complexes [33,34]. As described in the Manufacturing section, the NAPs are retained in some of the BoNT/A products and removed in others.

**Figure 1:**
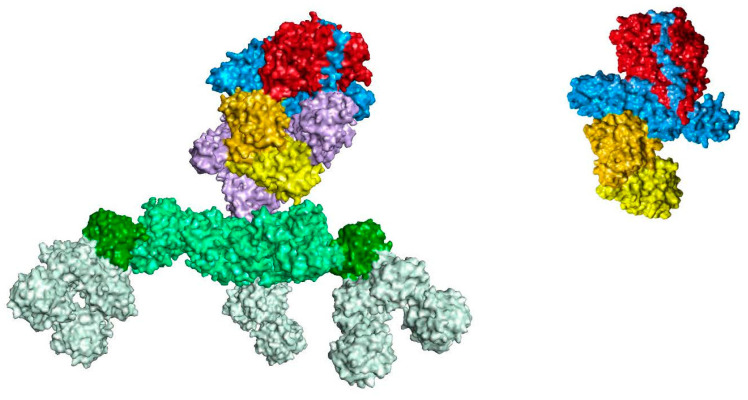
Structure of the BoNT/A LL-progenitor toxin complex (PTC; (**left**)) and the BoNT/A neurotoxin protein component (**right**). The complex shown here comprises the neurotoxin protein component (red/blue/yellow/gold), the non-toxin non-hemagglutinin protein (NTNH; purple), and several hemagglutinin (HA) proteins (shades of green). Images created by Lance Steward (AbbVie/Allergan Aesthetics) with Discovery Studio 2017 R2 (BIOVIA, Dassault Systèmes). Neurotoxin component image based on PDB ID 3BTA; Lacy et al. [44]. LL-PTC based on PDB IDs 4LO4, 4LO7, 4LO8, 4LO0 (RCSB.org; accessed on 29 February 2024); Lee et al. [45] and PDB ID 3V0A (RCSB.org; accessed on 29 February 2024); Gu et al. [31]).

*Clostridium botulinum* strains produce seven classical immunologically distinct serotypes of BoNTs, referred to as types A through G [35,36,37]. Various strains produce different BoNT serotypes, as well as PTCs of different sizes [32]. In 1946, the highest-molecular-weight complex of type A was reported to be ~900 kDa, calculated based on analytical centrifugation [38]. Later studies identified different-sized BoNT/A PTCs as the medium (M) ~300 kDa complex, large (L) ~500 kDa complex, and the extra large (LL) ~900 kDa complex based on gel filtration chromatography and sucrose density gradient centrifugation [39,40]; the BoNT/A 900 kDa complex has also been reported using size exclusion high-performance liquid chromatography [41]. The M-PTC is made up of BoNT and NTNHA, and the L- and LL-PTCs are formed by the association of various HAs (Figure 1). Some serotypes do not have the HA genes (BoNT/E and/F) and may only form the M-PTC [32]. The NAPs play various roles in BoNT’s activity, including protecting the neurotoxin from degradation [42] and potentially reducing exposure to the immune system [43]. Additional actions of the NAPs are described in a subsequent section (Role of NAPs in the Pharmacodynamic Action of BoNTs).

### 2.2. Mechanism of Action

For all BoNT serotypes, the ~150 kDa neurotoxin component is made up of two protein chains: a ~50 kDa light chain and a ~100 kDa heavy chain. These chains are linked by a disulfide bridge [46]. Specific locations or domains within the ~50 kDa light chain and a ~100 kDa heavy chain of the neurotoxin component mediate different aspects of the BoNT multi-step mechanism of action, which have inspired the moniker “modular nanomachine” [47].

The overall mechanism of action involves binding to nerve terminals, internalization into the neuron, translocation of the light chain, and cleavage of one or more proteins in the SNARE (soluble N-ethylmaleimide sensitive factor attachment protein receptor) complex that mediates vesicular fusion with the plasma membrane, resulting in inhibition of neurotransmitter release from the neuron.

**Figure 2:**
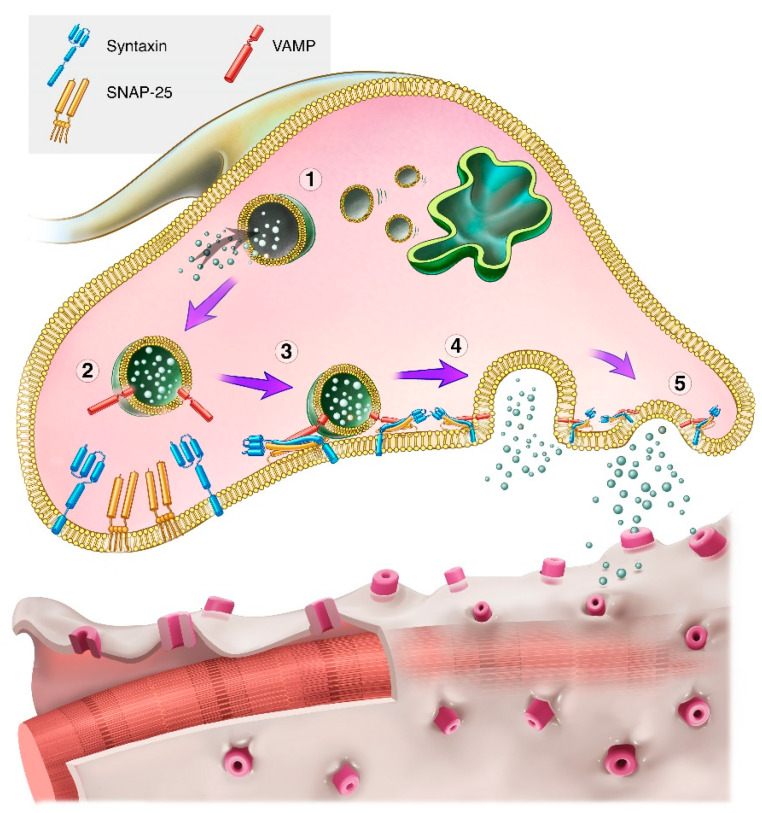

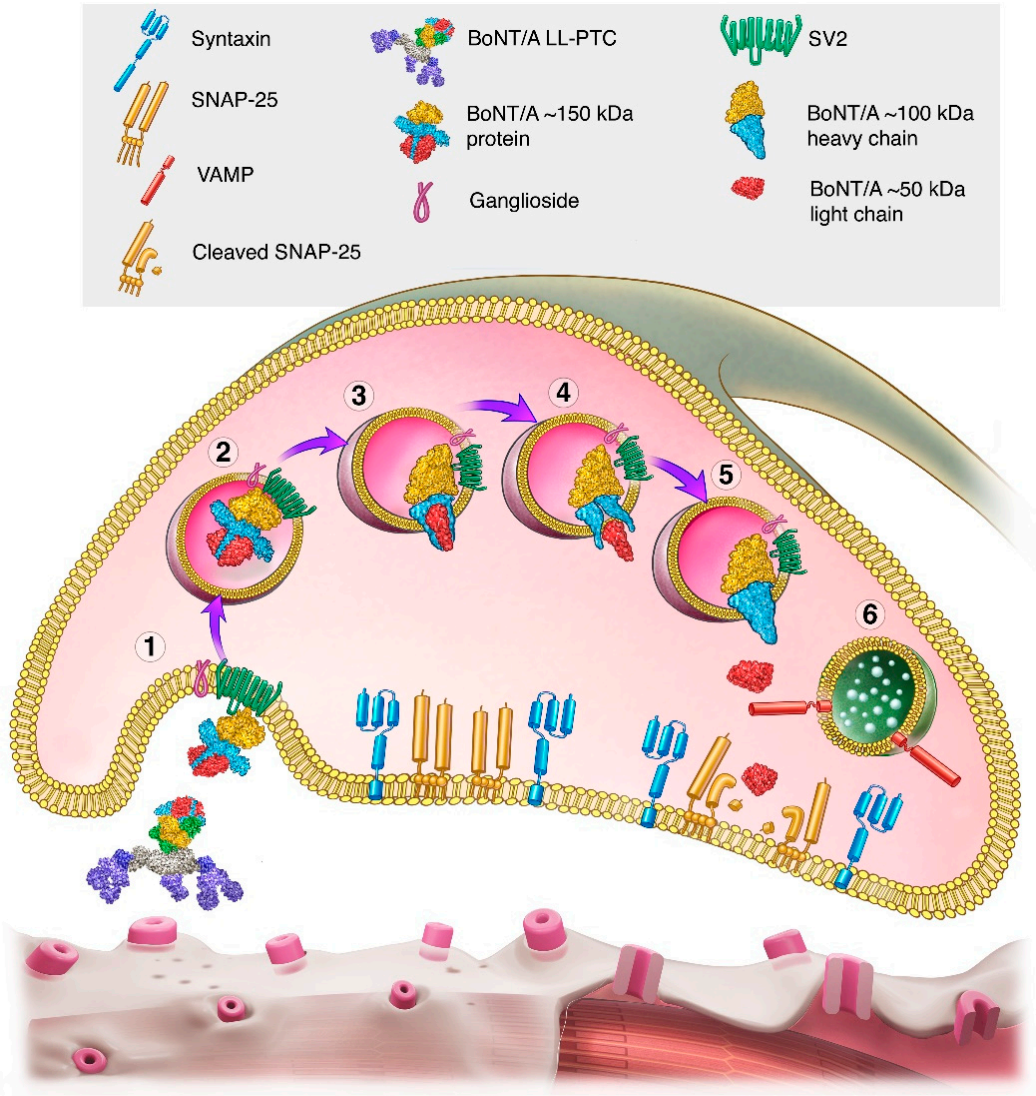
Mechanism of action of BoNT/A. The **top** panel shows fusion of cholinergic synaptic vesicles with the motor nerve terminal membrane in the absence of BoNT/A. Vesicles bud off the early endosome and are loaded with acetylcholine (1). The vesicle approaches the nerve terminal membrane (2), where the SNARE proteins in the vesicle membrane (VAMP; red) and neuronal membrane (syntaxin—blue; SNAP-25—gold) assist with vesicle docking and fusion (3). Acetylcholine is released into the synaptic cleft (4) where it binds to cholinergic receptors on motor neurons (5). The **bottom** panel shows the mechanism by which BoNT/A inhibits cholinergic neurotransmission. BoNT/A binds to gangliosides and the protein SV2 in the nerve terminal membrane (1). BoNT/A is then internalized into the neuron via receptor-mediated endocytosis (2). The BoNT/A heavy chain translocates the light chain of the protein across the synaptic vesicle membrane into the cytoplasm (3–5), where the light chain functions as a zinc-dependent protease, cleaving SNAP-25 and preventing vesicle fusion and hence acetylcholine release (6).

The mechanism of action of BoNT/A has been well studied and characterized. The first step involves dual binding of the C-terminal portion of the BoNT/A ~100 kDa heavy chain to low affinity gangliosides (lipid-carbohydrate molecules) on the surface of nerve terminals and to a higher-affinity synaptic vesicle protein, SV2, that becomes accessible during vesicular neurotransmitter release (Figure 2) [33,48].

After binding, BoNT/A is internalized into nerve cells via receptor-mediated endocytosis, where it temporarily resides within vesicles. The N-terminal portion of the BoNT/A heavy chain translocates the ~50 kDa light chain of the protein across the vesicle membrane (Figure 2) [49]. The disulfide bridge is then reduced, enabling the release of the light chain into the cytosol [46] where the ~50 kDa BoNT/A light chain cleaves synaptosomal associated protein-25 kDa (SNAP-25)—part of the SNARE complex.

Cleavage of SNAP-25 inhibits synaptic vesicle fusion with the neuronal membrane, thereby inhibiting vesicular neurotransmitter release such as occurs at the neuromuscular junction. It also inhibits other cellular processes that require synaptic vesicle fusion with membranes, including the insertion of protein receptors and channels from the vesicle into the membrane [50].

Although the general mechanism of action of BoNTs is well characterized, several detailed questions remain, such as the specific mode of endocytosis responsible for neurotoxin internalization (e.g., clathrin-mediated endocytosis, ultrafast endocytosis, and/or activity-dependent bulk endocytosis) and the specific localization of the light chains of various BoNT serotypes following translocation across the endocytotic vesicle membrane (e.g., continued association with the vesicle versus diffusion within the cytosol) [51].

In addition to its action on motor neurons, BoNT/A inhibits the release of pain-related peptides such as substance P and calcitonin gene-related peptide (CGRP) from sensory neurons [52,53]. BoNT/A further prevents plasma membrane trafficking of transient receptor potential (TRP) receptors, which are important in pain [54,55].

Additionally, BoNT/A binds to fibroblast growth factor receptor 3 (FGFR3) in motor neurons [56] and increases FGFR3 dimerization, a marker of ligand–receptor binding [57]. The contribution of FGFR3 binding to the actions of BoNT/A requires further investigation.

### 2.3. Serotypes

Although all BoNT serotypes exhibit the same general mechanism of action, their specific features and actions differ, which influence their clinical properties as described later. The serotypes have related but non-identical primary structures (amino acid sequences) [58], which determine secondary (local protein folding), tertiary (overall 3-dimensional structure), and quaternary structures (arrangement of protein chains) that are essential for biological activity.

**Figure 3:**
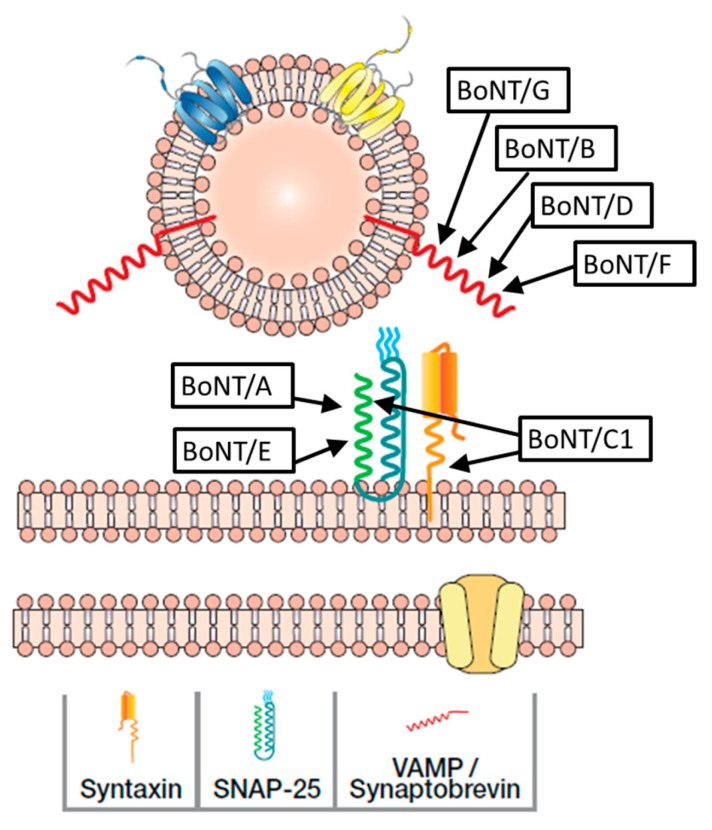
SNARE cleavage sites of different BoNT serotypes. BoNT serotypes B, D, F, and G all cleave VAMP/synaptobrevin at different sites. BoNT serotypes A, C1, and E all cleave SNAP-25 at different sites. Serotype C1 also cleaves syntaxin. Image modified from: Burstein et al., 2014 [61].

BoNT serotypes have different binding affinities for specific gangliosides on the nerve membrane, and the synaptic protein receptor varies by serotype [59]. For instance, BoNT/A binds primarily to synaptic vesicle protein 2C (SV2C), BoNT/E to SV2A and SV2B, and BoNT/B and/G to synaptotagmin [59]. BoNT/C1 appears to lack a protein receptor, instead binding to two gangliosides to mediate cell entry [60]. Inside neurons, each BoNT serotype cleaves a unique point on one or more SNARE proteins, resulting in the generation of different sized protein fragments (Figure 3) [33]. For instance, like serotype A, BoNT serotypes C1 and E also cleave SNAP-25, but at different sites than type A. Serotypes B, D, F, and G cleave vesicle associated membrane protein (VAMP)/synaptobrevin at specific sites; type C1 also cleaves syntaxin. Readers are referred to several expert reviews for details on mechanism of action [33,49].

### 2.4. Role of NAPs in the Pharmacodynamic Action of BoNTs

BoNT/A is naturally expressed in *Clostridium botulinum* as PTCs, with the complex conferring a thermodynamically stable structure [62] that protects the 150 kDa neurotoxin in harsh environments [63,64]. The NAPs have been shown to protect BoNT from proteolysis [42] and alter the secondary structure of neurotoxin conformation [62].

Emerging evidence suggests that the NAPs may play a fundamental, and intracellular, role in BoNT pharmacology. In particular, HA34, the most abundant HA in the BoNT/A LL-PTC [41], binds to the neurotoxin with a KD of 0.1–0.4 µM [65,66]. HA34 itself has been shown to increase BoNT/A endopeptidase activity in vitro and in synaptosomes [65]. In a comprehensive study in human bronchial epithelial cells, HA34 increased the kinetics of BoNT/A binding to the cell surface and trafficked with BoNT/A into the same intracellular vesicles [66]. HA34 has also been demonstrated to bind to cell-expressed carbohydrates [67] and synaptotagmin II (a synaptic vesicle protein and calcium sensor that is also the protein receptor for BoNT serotypes B and G in presynaptic axon terminals) [68].

In 1970, prior to the advent of therapeutic BoNTs, Carl Lamanna, Leonardo Spero and Edward Schantz reported in vivo preclinical experiments evaluating the biology of the ~900 kDa BoNT complex and 150 kDa isolated neurotoxin [69]. They assessed the time-to-death for both compounds when administered to mice intravenously and intraperitoneally. Their results demonstrated that the time-to-death dose response curves were neither overlapping nor parallel for either injection route, leading them to conclude that “these findings preclude a rapid conversion of the large to the small molecule under physiological conditions, but they are consistent with the hypothesis that the time the toxin takes to escape from body fluids to reach specific receptor sites is influenced by molecular dimensions and the related property of diffusion rate”. In a post hoc analysis, Allergan demonstrated that the biologic effects reported by Lamanna et al. were statistically different (Allergan Data on File).

Overall, the in vitro and in vivo preclinical studies support the concept of the neurotoxin complex as a critical biological component in the pharmacotherapy of BoNTs.

## 3. Transforming BoNTs into Medications

Given that BoNTs are large, complex biological products, transforming them into medications is more involved than for conventional small-molecule drugs. Small-molecule drugs are produced via a series of chemical reactions and their structures can be fully defined, which allows generic versions to be produced. In contrast, biological products such as BoNTs are produced by living organisms and then manufactured into medicines via complex and highly controlled processes during which they are subject to post-translational structural modifications that lead to intrinsic heterogeneity [70]. The manufacture of BoNT complexes is even more challenging because of the multiple proteins involved and protein–protein interactions. For these reasons, generic biologics are not possible.

Given that biological medications cannot be generics, the term biosimilars is used to describe biological medications deemed highly similar, but not identical to, the original innovator (reference) product, and that show no clinically meaningful differences in terms of safety, purity, and potency [71]. Notably, the concept “clinically meaningful” is notoriously difficult to define, particularly as it depends on the perspectives of different stakeholders (e.g., patients, caregivers, insurers, etc.) [72]. Although some of the BoNT/As in Table 1 have similarities, there are currently no BoNT biosimilars. For this reason, each BoNT product is referred to in the United States by a unique United States Adopted Name (USAN) nonproprietary name (Table 1). The following text outlines the manufacturing process for BoNTs, noting the variations at each step that can affect the nature of the final products.

### 3.1. Bacterial Strain

The manufacturing process for each commercial BoNT product is distinct and proprietary, beginning with the master cell bank containing the *C. botulinum* bacterial strain. The single commercial BoNT product based on the B serotype is produced from the *C. botulinum* type B Bean strain [13]. Some of the available BoNT type A products are based on “a Hall strain” as noted in the next paragraph (e.g., onabotA, incobotulinumtoxinA (incobotA), abobotulinumtoxinA (abobotA), daxibotulinumtoxinA (daxibotA)) [3,7,9,73], whereas others are based on different strains (e.g., letibotulinumtoxinA (letibotA): CBFC26 strain) [74,75].

In the early 1900s, bacteriologist Ivan Hall isolated and preserved a number of different *C. botulinum* strains from several sources [76]. and eventually distributed them to various academic institutions where they were sub-cultured. These strains became known as “the Hall strain”, even though they are not identical [77,78,79]. A comparison of four different bacterial strains producing BoNT type A, three of which were identified as Hall strains, found differences in neurotoxin gene sequence, gene content, and genome arrangement [78]. Even minor differences in the amino acid sequence can substantially alter in vitro and in vivo properties of BoNTs, including onset and duration of effect [80,81].

### 3.2. Fermentation

*C. botulinum* bacteria produce BoNT when they are fermented under appropriate conditions. Fermentation is an anaerobic metabolic process used by bacteria and yeast to generate energy for cell growth; fermentation is best known as the process by which yeast produce wine from grapes and beer from grains. In the case of *C. botulinum* bacteria, neurotoxin is produced when the bacteria are cultured and maintained under conducive conditions. Each manufacturer uses its own proprietary fermentation method, including the constituents of the fermentation media, which includes nutrients such as carbon, nitrogen, and hydrolysate (amino acid) sources. The growth conditions and duration of fermentation may vary. Fermentation conditions such as glucose concentration and temperature will affect production of BoNT/A [82] and may be expected to have different quality attributes and yield between manufacturers.

### 3.3. Purification

The next step in BoNT manufacturing is purification of the proteins from the fermentation broth. The purification methods used for each product are proprietary and contribute to the specific characteristics of the drug substance (e.g., complex size, protein configuration). Purification is accomplished by crystallization for onabotA [83] and chromatography for many other BoNTs, including abobotA [84], incobotA [85], rimabotulinumtoxinB (rimabotB) [13,82], daxibotA [73], and letibotA [74]. However, even within a given purification method such as chromatography, the specific methods used for each product can differ, such as processing reagents, etc. Given that the purification methods are proprietary, it is not possible to compare specific procedures across manufacturers. Notably, manufacturers intentionally design their procedures to retain some, all, or none of the NAPs (Table 1; Figure 1).

Purification results in the drug substance—an active ingredient that is intended to furnish pharmacological activity [86]. For BoNT products, NAPs are part of the drug substance if they are retained during purification. In vitro, NAPs increase the stability of the ~150 kDa neurotoxin component at a range of physiologically relevant temperatures and pH values [87] and may reduce exposure to the immune system [43]. Additional roles of the NAPs are described in a previous section (Role of NAPs in the Pharmacodynamic Action of BoNTs). The drug substance is stored in the manufacturer’s drug substance-specific formulation and aliquots are subsequently used in the manufacture of drug products.

The manufacturing processes described to this point are designed to obtain BoNT proteins, with or without NAPs, that retain their secondary, tertiary, and quaternary structures (when present). As with all proteins, even small changes in the manufacturing process can lead to changes in protein biochemistry or structure, impacting function and biological activity [88]. This is not merely theoretical: manufacturing changes have led to unexpected and consequential alterations with protein therapies including alglucosidase alfa (upscaling production resulted in glycosylation differences that necessitated a new biological license application) [89] and a human growth hormone product (increased antibody formation occurred due to host cell protein contamination) [90,91]. Preservation of protein biochemistry and structure is therefore one reason that the manufacturing processes for biological proteins must be strictly controlled and monitored. In-process testing throughout the manufacturing process is utilized to characterize protein integrity, purity and activity during processing.

### 3.4. Unit Testing Procedures

For chemically synthesized drugs such as acetaminophen, amounts are measured in mass or weight in milligrams, micrograms, or nanograms. However, weights are not adequate measures of potency of BoNTs because of the complexities inherent in large proteins and their manufacture. The clinically relevant measure for BoNTs is not the weight of the substance present (e.g., in a vial or syringe) but rather the ability of that substance to affect biological processes. As such, BoNTs are measured in units of biological activity that are not interchangeable among different products. The specific features of unit testing, including differences among manufacturers’ assays that lead to potency differences among BoNTs, are described in a subsequent section (Botulinum Neurotoxin Potency).

### 3.5. Excipients and Formulation—Generating the Drug Product

The final step in the manufacturing process of BoNTs is fashioning the drug substance into a form that can be used by clinicians. The finished dosage form of a medication is the drug product [86].

BoNT drug products include the drug substance along with excipients. Excipients are substances that are appropriately evaluated for safety and intentionally added to the drug substance [92], to maintain integrity/stability and enable delivery of the drug substance (Table 1).

Several currently available solid BoNT preparations (onabotA, abobotA, incobotA, prabotA) include large proteins such as albumin as excipients. In the initial formulation of onabotA for clinical use, albumin was used to help ensure stability during the reconstitution process [83], increase the amount of physical substance in the vials, and help prevent BoNT from adhering to surfaces such as glass. The large protein, gelatin, is included in the BoNT/A products from Lanzhou and Microgen (Table 1). Sugars are also used as bulking agents to enhance product stability or provide structure in lyophilized preparations, such as the lactose in abobotA, sucrose in incobotA, maltose in Relatox^®^, and trehalose dihydrate in daxibotA.

Surfactants are excipients that help prevent large proteins binding to contact surfaces or reduce interfacial tension at the liquid/air interface (e.g., polysorbate-20, daxibotA; polysorbate-80, and liquid abobotA). BoNT products also contain a tonicity agent (e.g., sodium chloride, or trehalose) to help control osmolarity of the injected substance (i.e., prevent hypo- or hypertonicity).

The recently introduced daxibotA contains the proprietary cell-penetrating peptide RTP004. A study by Malmirchegini et al. found that the proprietary peptide prevented the neurotoxin from thermal aggregation in solution and adsorption to the vial surface [93]. Conversely, two groups reported that cell penetrating peptides did not have significant effects on the adsorption or aggregation of BoNT/A [94,95]. As such, the role of the proprietary peptide in daxibotA remains unclear.

A finishing process results in the final commercial form as solids or liquids. BoNTs formulated as solids require some method of drying to deliver the final drug product. AbobotA and letibotA are freeze-dried, and several other products (e.g., incobotA, prabotulinumtoxinA (prabotA) [in Korea]; BoNT/As from Microgen and Lanzhou) are lyophilized. Freeze drying and lyophilization (often used interchangeably) are processes in which the liquid is frozen and the ice evaporated under low pressure. OnabotA is vacuum dried, in which the liquid is removed under reduced air pressure without the freezing step.

The first liquid BoNT formulation to be approved in the US was rimabotB, which is formulated as a buffered solution of pH = 5.6 [14]. A liquid abobotA is approved in EU for glabellar lines, and additional liquid formulations are in development by multiple manufacturers (Table 1). Liquid formulations may reduce the time and burden associated with reconstitution in clinics, as well as the potential for medication errors. However, liquid formulations limit the ability to modify injected product concentration when needed to individualize patient care. Overall, the variety of BoNT product formulations provides options for clinicians.

### 3.6. Pre-Release Unit Testing

**Figure 4:**
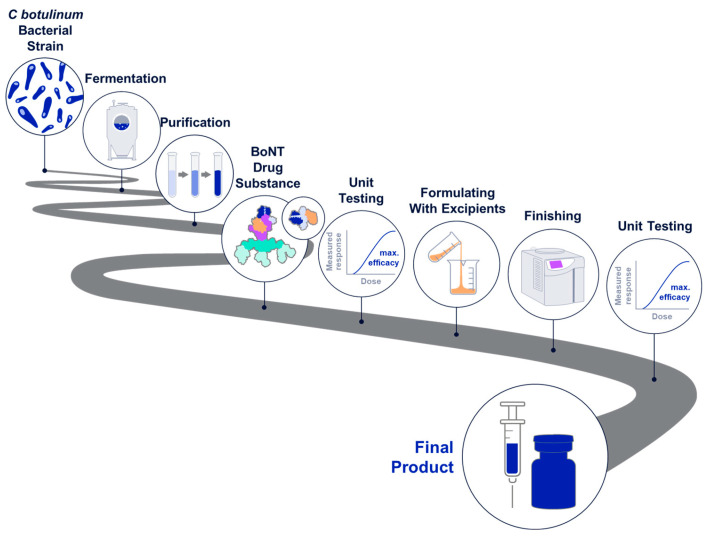
Overview of manufacturing process for BoNTs. This graphic shows the main steps of manufacture for BoNTs. Differences among products can occur at each step, as described in the text.

After compounding and finishing, BoNT products are tested for unit activity prior to release for clinical use (Figure 4). Pre-release assay conditions differ from those used for drug substance testing, as drug substance concentration and drug product excipients can influence assay performance [96]. The potential for interaction of unique excipients with LD50 and cell-based potency assays (CBPAs) adds a layer of complexity to the differences among BoNT product Unit assignment.

## 4. Botulinum Neurotoxin Potency

Potency in pharmacology is defined as the concentration or amount of drug needed to produce a defined effect [97,98]. As noted previously, potency for BoNTs is measured in units of biological activity rather than in weight. In biological activity assays, a number (unit) is assigned to a functional effect. For BoNTs, units of biological activity are specific to each product and manufacturer and are not interchangeable. The non-interchangeability of units is based on differences in unit testing procedures implemented by manufacturers, which includes differences in determining the potency of reference standards. Per the European Pharmacopoeia, potency of BoNT/A products for injection must be confirmed in a mouse model of toxicity or by in vivo/ex vivo methods validated with respect to the mouse LD50 assay (see next section on Definition of a Unit) [99]. Advances in technology have permitted the development of cell-based potency assays for BoNT potency testing while ensuring the pharmaceutical quality of the product (see Cell Based Potency Assays).

### 4.1. Definition of a Unit

The mouse LD50 (mLD50) has been the standard method for BoNT unit testing against which newer methods (e.g., CBPAs) are anchored. One unit is defined as the median lethal dose in mice following intraperitoneal (IP) injection. Mouse LD50 testing is performed under controlled conditions to promote consistency (e.g., animal strain, age, sex, diet, temperature, caging, season, liquid used to dilute the product, etc.) [14,100,101]. Although methods of mLD50 testing are standardized for a specific product, procedures vary between companies. For instance, all BoNT manufacturers use proprietary reference standards (see Potency Reference Standards section) and many use different assay protocols, including reconstitution agents, dilution schemes, etc., resulting in units that are not comparable or interchangeable. Indeed, different assays are needed to accommodate the formulation differences among BoNT products.

The influence of assay conditions on mLD50 outcomes has been studied by Sesardic and colleagues working at the National Institute for Biological Standards and Control in Potters Bar, Hertfordshire, United Kingdom [96,102]. Their studies found a differential effect of assay conditions on BoNT/A products from different manufacturers [96]. For instance, the strain of mice used and the addition of gelatin phosphate to the dilution buffer had a greater effect on the mLD50 values of some BoNT preparations than others [96]. For this reason, they concluded that mLD50 tests and the units obtained are specific to each BoNT manufacturer and only apply to each individual BoNT product. Sesardic and colleagues also found inter-and intra-laboratory variation in mLD50 values, that was improved by the use of a reference standard [102].

### 4.2. Potency Reference Standards

Potency reference standards are an important aspect of unit testing that renders BoNT units unique and non-interchangeable. Reference standards are certified materials or substances whose properties are sufficiently well established that they can be used for calibration of an apparatus, assessment of a measurement method, and assigning values to materials [103]. Potency reference standards are used to calibrate assays that measure the biological activity of BoNT. Each company creates and maintains its own proprietary potency reference standard that defines a unit and against which the potency of each BoNT lot intended for commercial use is measured and compared. Consequently, the units of each BoNT remain relative to each manufacturer’s specific proprietary qualified reference standard according to international guidelines. Small proteins such as insulin have international standards against which the potencies of products from different manufacturers are compared [104]. International reference standards are also available for some large proteins such as infliximab, but these standards are to be used only for each manufacturer’s quality control purposes and not to compare different products [105].

The non-interchangeability of units or unit doses among BoNT products is a critical clinical concept given the increasing number of BoNTs that are now commercially available or in development worldwide (Table 1). Non-interchangeability of units means that 100 units of one BoNT product are not the same and do not have the same potency as 100 units of another product because each BoNT is unique due to the differences in manufacturing process and units of each are determined by different assays and internal reference standards.

### 4.3. Cell Based Potency Assays

Although the mLD50 has been the basis of BoNT potency testing for decades, some BoNT manufacturers have sought to reduce animal use and develop methods that can accommodate high-volume testing. Today, proprietary CBPAs are increasingly used to assess potency of BoNTs in place of the mLD50 [7,9,106]. The potency reference standards used for these assays are qualified based on mLD50 tests and thus trace their lineage back to that test. For CBPAs to be approved by regulatory agencies, they must be rigorously developed and cross validated against product-specific mLD50 tests. Additionally, CBPAs must recapitulate all steps in the mechanism of action of BoNTs, including binding, internalization, translocation of the light chain, and SNARE protein cleavage [107].

**Figure 5:**
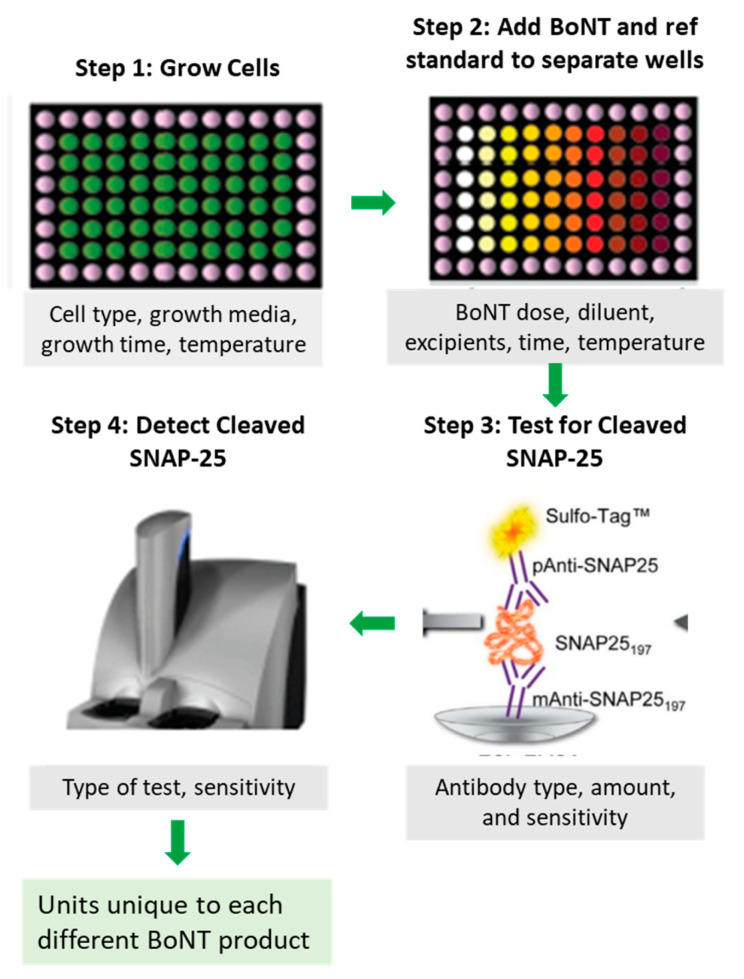
Factors that affect the development of cell-based potency assays (CBPAs). The development of CBPAs is influenced by many different factors, some examples of which are listed under each step. Variations in these factors influence assay performance. This graphic depicts the general steps in the Allergan/AbbVie CBPA. Modified from Rupp et al. 2021 [108] and Fernandez Salas et al. 2012 [106].

Development of a BoNT CBPA is a difficult undertaking due to the specificity and sensitivity required to detect the minute amounts of BoNTs in medicinal products. Like mLD50 tests, CBPAs are impacted by a large number of factors, including the (1) type of cells, (2) number of cells, (3) conditions/media used to grow and maintain cells, (4) treatment times, (5) incubation times, (6) diluent that maintains integrity of the BoNT sample and is compatible with cell viability, (7) antibody or other molecular reporters used to detect SNAP-25 cleavage, and (8) antibody amount (if antibodies are used) (Figure 5) [106]. These factors are optimized differently by individual manufacturers in their own proprietary assays. For example, differences in the sensitivity and specificity of antibodies against SNARE protein fragments used by different manufacturers can lead to more or less BoNT required to meet the definition of a unit in a specific assay.

### 4.4. Examples of Differences in Potency among BoNTAs

Given the differences in mLD50 tests, CBPAs, and reference standards described, it is expected that BoNT products would yield different potencies when compared at the same number of labeled units. This has been demonstrated in two recent studies in which onabotA displayed greater potency than incobotA [109] and prabotA [108] in the Allergan/AbbVie CBPA. In the CBPA, incobotA demonstrated reduced relative potency compared with onabotA, showing in a 1.3-fold difference [109]. A separate study that compared prabotA with onabotA in the CBPA also found a reduced relative potency of prabotA compared with onabotA, showing a 1.3-fold difference [108]. Thus, although incobotA and prabotA were labeled as 100 U and tested as 100 U in their own manufacturers’ potency assays, they measured less than 100 U when tested in the onabotA potency assay. This means that more than 1 U incobotA and prabotA are needed to achieve the same biologic effect as 1 U of onabotA.

### 4.5. No Fixed Dose Ratios

The lack of a standardized unit for BoNT products has led to attempts at defining dose conversion ratios. Preclinical studies conducted over the past several decades have demonstrated that dose ratios of BoNT products vary in different experimental models and for different outcome measures [110,111,112,113]. These preclinical studies, conducted under controlled conditions, demonstrate that there is no fixed dose ratio across the range of doses and no single dose ratio is accurate to compare BoNT products.

The preclinical findings are supported by clinical evidence, as shown in Table 2 and Table 3 for several BoNT products. Ratios of onabotA:abobotA doses in clinical studies have ranged from 1:1.2 to 1:13, with blepharospasm showing the most variability (Table 2). In cervical dystonia, hemifacial spasm, and spasticity, onabotA:abobotA doses have tended to be used at ratios ranging from 1:2.5 to 1:6 (Table 2). Ratios of onabotA:incobotA doses in clinical studies have ranged from 1:1 to 1:2.5, with the majority of ratios >1 and none of the ratios <1 (Table 3).

## 5. Botulinum Neurotoxin Dose Response

Dose response is a basic principle of pharmacology in which responses to medications are evaluated in relation to increasing doses. Due to the nature of biological systems, responses often increase to a finite point beyond which they do not increase further regardless of the increase in dose [141]. At extremely high doses, therapeutic responses can even decline, for example, due to toxicity, adverse events, or other factors related to the system under study [142].

**Figure 6:**
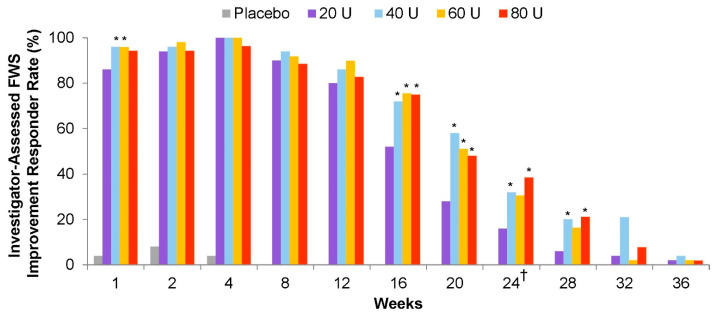
Dose response of onabotA duration. Proportion of responders at each visit (subjects with a ≥1-grade Facial Wrinkle Scale [FWS] improvement from baseline at maximum frown), assessed by investigator. Data are from all randomized and treated subjects with a baseline and at least 1 postbaseline efficacy assessment, only in the double-blind phase. * *p* < 0.05 vs. onabotA 20 U by the Cochran Mantel-Haenszel test stratified by center. ^†^ Primary time point. Figure reproduced from Joseph et al., 2022 [146] (Creative Commons CC by NC; https://creativecommons.org/licenses/by-nc/4.0/).

Like most medications, BoNTs exhibit a dose response relationship. As doses are increased, more SNAP-25 is cleaved [105,143], leading to greater inhibition of neurotransmitter release [143,144]. BoNT dose response has been documented in non-human preclinical studies in which a wide range of doses can be evaluated [145], as well as in clinical studies evaluating a narrower range of doses for many of the BoNT products (e.g., onabotA [146], abobotA [147,148], incobotA [149], daxibotA [150]; see Figure 6 for an example). In the dose-ranging glabellar line studies, efficacy/duration responses appear to plateau at the highest doses, representing the peak pharmacological effect and/or duration [146,150], an observation consistent with typical dose–response kinetics characterized by a “ceiling effect” or maximal attainable response [151]. Conversely, in several studies that evaluated only two doses or two dose ranges of incobotA [9] and daxibotA [152] for cervical dystonia, responses appeared to be similar across doses. In these studies, it is unclear whether the data plateaus represent a ceiling effect or whether higher doses would produce greater efficacy or longer duration.

Dose response can be assessed not only for efficacy and duration, but also for adverse events, reflecting safety. In preclinical studies, dose response curves generated for different variables have permitted comparison of safety margins among BoNTs. In such studies, the safety margin of onabotA has been reported to be significantly higher than those of abobotA and rimabotB [110,111]. The difference in preclinical safety margins is supported by clinical studies that have found differences in adverse events among these BoNT products [121,153].

## 6. BoNT Onset of Effect

In clinical use, BoNT/A has been reported to exert noticeable effects within 24 h of intramuscular injection [154,155], with >90% of individuals treated for facial lines reporting effects within 3 days [156]. The onset of BoNT/B is also evident within 1–3 days of intramuscular injection [157,158]. Type E has been reported to have an onset of 1 to 2 days in humans and a more robust effect with a recombinant BoNT/E than abobotA at earlier timepoints [159]. The onset of efficacy (at least 2-grade FWS improvement from baseline) of an investigational BoNTE product (trenibotulinumtoxinE) was demonstrated at 8 h after administration, which was the earliest time point assessed [26].

The faster onset of type E has been suggested to relate to a cellular mechanism, in which it is translocated more rapidly from the endosome into the cytoplasm of neurons where it exerts its action (see Figure 2) [160,161]. The more rapid translocation may be due to BoNT/E’s structure, which leads the protein to adopt a position in the endosomal vesicle that permits faster entry into the neuronal cytoplasm [162]. BoNT/E’s more rapid onset of action and shorter duration of action (see next section) may be desirable for certain clinical uses such as previewing aesthetic results and inhibiting muscle contractions immediately prior to or following surgical procedures.

## 7. BoNT Efficacy and Duration of Action

In clinical pharmacology, efficacy refers to a medication’s therapeutic effect and duration broadly refers to the length of the therapeutic effect. Several general concepts may be considered in clinical scenarios relevant to efficacy and duration of action of BoNTs: (1) intrinsic factors attributable to the products themselves (e.g., serotype, formulation, and unit potency); (2) peripheral neuron type (e.g., motor, autonomic, and nociceptors); and (3) study-level differences (e.g., patient population, doses, injection paradigm, duration assessments, etc.) (Table 4).

### 7.1. Influence of Intrinsic Product Differences

#### 7.1.1. Serotype/Subtype

The various BoNT serotypes bind to different receptors, which include synaptic vesicle proteins and gangliosides (see Mechanism of Action section) [59]. The distribution and affinity of receptors influence the activity of BoNTs [59]. Within a given BoNT serotype, different subtypes can exhibit different potencies, as has been documented in laboratory models [80].

The fundamental differences in duration among BoNT serotypes are driven by intracellular biology. Duration differences among BoNT serotypes have been well documented in isolated nerve cell cultures [143,163], which are studied under controlled conditions and permit dose response assessments. In such studies, serotypes B and E exhibit a shorter duration than serotype A [143,163]. The shorter durations of serotypes B and E compared with type A have been confirmed in human studies [26,153,159,164].

The mechanism underlying the long duration of type A has been under investigation for several decades and likely involves multiple factors, including (1) a longer persistence of the type A light chain in nerve endings [143,163] and (2) the site at which it cleaves SNAP-25 (synaptosomal associated protein-25 kD) [165].

##### Persistence of Type A Light Chain

As described in the mechanism of action section of this paper, the light chain of the BoNT protein translocates across the endosomal membrane into the cytoplasm to cleave one or more SNARE proteins (Figure 2). In the cytoplasm, the type A light chain has a long-lasting effect that is not observed with types B or E because it is not degraded as quickly [143].

Although the reason for this slower degradation is not yet fully established, it has been suggested that different intracellular light chain localization may make them differentially accessible for degradation [166]. The light chains of the various serotypes congregate at different regions inside the cell: the type A light chain associates with cytoskeletal proteins (septins) and localizes to the neuronal membrane; the type B light chain is dispersed throughout the neuron; and the type E light chain is located mainly in the cytosol [166,167,168].

The type E light chain is rapidly marked for degradation by the ubiquitin–proteasome system—one of the main protein degradation systems in the cell—via the attachment of ubiquitin proteins that mark it for elimination [169]. The type A light chain is also ubiquitinated; however, the BoNT/A light chain recruits and directly binds a deubiquitinating enzyme (VCIP135), which removes ubiquitin [170]. This antagonistic relationship between ubiquitination and deubiquitination ultimately slows the protein’s degradation [170]. Another deubiquitinating enzyme (USP9X) may also indirectly contribute to prolonging the longevity of BoNT/A in cells [170,171]. Either or both mechanisms (light chain localization to the membrane and ubiquitination/other intracellular clearance pathways) may be responsible for the slower degradation of the type A light chain and its continued protease activity.

##### Site of SNAP-25 Cleavage

Studies suggest that at least one additional mechanism may contribute to the longer duration of BoNT/A than/E, namely, the site at which it cleaves SNAP-25. Although BoNT/A and/E both cleave SNAP-25, they target different sites: BoNT/E cleaves SNAP-25 between amino acids 180 and 181, whereas BoNT/A cleaves SNAP-25 between amino acids 197 and 198 [172]. The larger SNAP-25 protein fragment left by BoNT/A (i.e., amino acids 1-197) can continue to incorporate with VAMP and syntaxin into the SNARE complex [173,174]. The SNARE complex with the truncated SNAP-25 fragment generated by BoNT/A cleavage enables vesicle docking at the membrane but does not permit fusion and therefore is not functional—it does not mediate exocytosis [175,176]. Through this mechanism, the truncated SNAP-25 may exert a “dominant negative” effect, preventing any remaining full SNAP-25 protein from forming SNARE complexes and interfering with SNARE functions [33,174]. In contrast, the truncated SNAP-25 fragment generated by BoNT/E cleavage (i.e., amino acids 1–180) is smaller and not does incorporate into SNARE complexes [177].

#### 7.1.2. Formulation and Unit Potency

Even among BoNT products of the same serotype, other product factors such as formulation and units influence efficacy and duration. For instance, in preclinical studies where the effects of other variables are minimized, some BoNT/A products exhibit different durations at the same labeled unit doses [113]. In clinical studies, unit doses of BoNT/A products needed to achieve a comparable duration can vary several-fold [121,124].

### 7.2. Influence of Peripheral Neuron Type

Duration of effect may depend on the indication and type of peripheral neurons present in the tissue. Following injection into skeletal muscles at labeled doses, BoNT/A products generally exhibit durations of approximately 3 to 4 months in placebo controlled studies [4,7,9,11]. When injected into smooth muscles innervated by autonomic cholinergic nerves for the treatment of incontinence due to neurogenic detrusor overactivity, onabotA (200 U) shows a mean duration of 42 to 48 weeks [4]. In overactive bladder, the median time to re-treatment for onabotA (100 U) is 19 to 24 weeks [4]. Following intradermal injection into the axilla, where glands receive autonomic cholinergic innervation, onabotA (50 U) shows a mean duration of 28.7 weeks [4], with more than 22% of patients reporting a response for at least 52 weeks [178,179].

The long duration of BoNT/A in the bladder has been attributed to a lack of axonal sprouting following injections [180], although this explanation remains theoretical, particularly in view of the preclinical evidence that neuronal sprouts from the alpha motor neurons innervating skeletal muscle are relatively ineffectual for functional contractions [181]. In tissue from individuals with palmar hyperhidrosis, initial axonal sprouting was observed 3 months following BoNT/A injection but was not linked to chemodenervation and was not followed by reinnervation of the sweat gland [182]. The authors interpreted this to suggest that an imbalance between chemodenervation and sprouting in palmar tissue may underlie the long duration observed. This is consistent with other work showing that early neuronal sprouts are poorly functional in terms of neurotransmission [181].

### 7.3. Influence of Study-Level Factors

In addition to differences in the intrinsic properties of BoNTs and their interactions with various tissue types, differences among BoNTs can be observed at the clinical study level that may or may not reflect differences in the underlying intrinsic properties of the individual medications. Clinical study-level variables can affect conclusions about the comparability of BoNTs and include the patient population studied (e.g., indication, inclusion/exclusion criteria), differences in doses and treatment paradigms, assessment methods (e.g., rating scales and raters), and follow-up timepoints (Table 4).

#### 7.3.1. Patient Population

Various BoNT/A products are indicated for multiple conditions that differ in their complexity. For instance, several BoNT/A products are indicated for glabellar lines, a common condition that is treated by injecting a defined set of facial muscles. Some BoNT/A products are also indicated for spasticity, a condition that varies in its presentation, with patients experiencing involvement of different combinations of, for instance, finger, elbow, shoulder, ankle, and other limb flexors and extensors. Treatment of spasticity must be individualized, with different doses of each product selected and administered to the muscles involved, in support of a pre-defined treatment goal. Assessing efficacy and duration in complex conditions like spasticity can be challenging and it is difficult to compare between studies.

Even among patients with the same medical or aesthetic condition, variations in severity and muscle mass may affect BoNT pharmacodynamics and consequently efficacy and duration. Weight can be a factor in some studies (e.g., pediatrics) due to weight-based dosing guidelines.

In designing clinical trials with the intent of identifying a responsive population, studies may focus on patients who are the most likely to benefit from treatment. The responsive population may be refined over time as lessons are learned from clinical studies and practice. Consequently, efficacy and duration in early studies may appear to be lower than in later studies in which less responsive groups of patients have been excluded. This is evident in the cervical dystonia literature in which early studies of onabotA and abobotA allowed enrollment of patients with predominant retrocollis [183] and anterocollis [184], which are more difficult to treat with BoNTs due to their complexity and the involvement of deeper or poorly accessible muscles [185,186]. More recent studies with incobotA [187] and daxibotA [152] excluded these patients. This natural progression in trial populations means that later studies can be enriched with patients who are more likely to respond to treatment and may result in more favorable outcomes such as a greater improvement in scores or a longer duration of effect.

Another example in which the patient population can influence efficacy and duration outcomes can be found in the glabellar lines literature, with some studies including a higher population of subjects with moderate as opposed to severe lines at baseline. An outcome of a none or mild on a glabellar lines rating scale will be easier to achieve over a longer period in subjects with less severe lines at baseline. Other endpoints such as a 2-point improvement on a glabellar lines rating scale may favor subjects with severe lines at baseline. Potential bias can be avoided by enrolling an equal proportion of subjects with moderate and severe lines at baseline, but different enrollment strategies may be used based on the experimental question.

#### 7.3.2. Dose Differences

Units of BoNT products are not interchangeable and it can therefore be difficult to determine the comparability of doses across studies. Dose differences are relevant because efficacy and duration increase with dose up to a maximal point (see Dose–response section). As noted previously, in placebo controlled studies at labeled doses, BoNT/A products generally exhibit durations of approximately 3 to 4 months following injection into skeletal muscles [4,7,9,11]. In a dose-ranging study of daxibotA for the treatment of glabellar lines, the median durations of a ≥1-point improvement from baseline on the Investigator Global Assessment-Facial Wrinkle Scale were 20.0 weeks with 20 U, 23.6 weeks with 40 U, and 20.9 weeks with 60 U [150]. The 40 U dose of daxibotA was selected for phase 3 development in glabellar lines because it had the most favorable risk:benefit profile [150]. Increasing the doses of several other BoNT/A products increases their durations of action, as documented for glabellar lines (see Dose Response section) [146,147,149], and cervical dystonia [188], with the exception of the 150 kDa products incobotA [9] and daxibotA [152] at the doses tested in registration clinical trials for cervical dystonia.

#### 7.3.3. Treatment Paradigms

Prospective clinical studies typically have highly structured protocols that may not reflect clinical practice. This includes treatment paradigms, which are often standardized for the particular BoNT and indication under study.

Given that units of BoNT products are not interchangeable and each product has different physicochemical properties due to its unique formulation, the doses and muscles injected can vary from one product to the next. For instance, the doses, numbers, and locations of recommended muscles and injection sites in upper limb spasticity are different for onabotA [4] and abobotA [7], making it difficult to design studies with protocols optimized for each. These differences are confirmed by a recent analysis of real-world studies in upper limb spasticity that found that the two products were not only injected at different doses, but also at different ratios of doses per muscle, indicating that the products are not used at a set dose ratio in clinical practice [189]. This study of adult post-stroke patients also found that a higher number of muscles were injected with onabotA than abobotA [189]. These differences are underscored by the different injection paradigms outlined in the product labels, which for onabotA includes 13 potential muscles to be injected in upper limb spasticity [4] and for abobotA includes 9 potential muscles [7]. Given these differences, comparing the products in spasticity using the same injection paradigm may favor one product over another. For instance, a recent study uses the same injection sites for both onabotA and abobotA in upper limb spasticity, limits injection sites to 5 pre-identified muscles for both, and avoids the finger flexors [190].

#### 7.3.4. Assessments of Efficacy and Duration

Efficacy and duration of response can be assessed using many different methods that vary from statistical estimates to more clinically relevant improvement that is important to patients. Efficacy may be expressed in terms of responders at various timepoints throughout the study, which can then be used to describe duration. Different assessments and definitions of response often give different estimates of efficacy and duration, underscoring the importance of carefully considering the assessments that underlie the reported outcomes in each individual study.

The glabellar lines literature provides an example of the many different definitions that have been used to define a responder, which in turn affects the estimated duration that is based on responder rate. For instance, a study of abobotA specified response as days to return to grade 2 (moderate) or 3 (severe) on a 4-point categorical scale [147], whereas a study of incobotA specified time to return to baseline [191]. Studies of incobotA [149], daxibotA [150], and onabotA [192] specified at least a 1-point improvement from baseline as the definition of response.

Duration can be measured as point estimates of responder rates at study visits or through statistical analyses such as the Kaplan–Meier method. In the aforementioned glabellar lines studies, responder data were estimated using the Kaplan–Meier method, a time-to-event analysis. In the context of BoNT duration estimates, the defined event is loss of response based on the definition of a responder used in the study. The Kaplan–Meier plots for BoNT durations are graphical representations that use horizontal lines to show the probability of maintaining response and vertical lines to show loss of response. Duration in Kaplan–Meier analyses is often reported as the median—the timepoint at which the likelihood of continuing to respond (i.e., not experiencing the event—loss of response) is 50%. Like all measures, the Kaplan–Meier analysis has some limitations, and its use in the context of BoNT duration has been questioned by the FDA because different criteria may be used to define the endpoint event [8]. Another limitation of the Kaplan–Meier in the context of BoNT duration studies is that it only includes subjects who were considered responders at a given timepoint early in the study (e.g., 4 weeks post-treatment) and excludes non-responders. The method is additionally limited by the frequency of study visits, such that subjects are assumed to be responders until the visit date at which they did not meet the response criteria. This can overestimate the duration of response for subjects who lose response between visits because they are considered responders until their next visit. Additionally, it is important to verify that the definition of clinical response applied to the duration analysis is the same as that used for determining efficacy. However, an important advantage of the Kaplan–Meier method is that it captures data from patients who left the study (known as censored), which are typically shown as tick marks or dots on the horizontal lines of the graph.

Different assessments of duration are also evident in the spasticity literature, making it problematic to compare across studies. For instance, time to re-treatment has often been used as an estimate of duration but can itself be defined in various ways. In clinical practice, patients typically request retreatment prior to returning to baseline and injection sessions are routinely scheduled a certain number of months apart. Thus, time to re-treatment does not necessarily measure continued clinical effect, even though it can be useful for some purposes. In the setting of a clinical trial, a more systematized framework is generally required for a structured analysis. In the phase 3 study of abobotA for upper limb spasticity, subjects qualified for re-treatment only when their scores on the modified Ashworth scale returned to baseline [193]. Other studies have required that subjects meet two assessment criteria instead of one, as in the case of a daxibotA phase 2 study for spasticity in which subject scores must have returned to baseline on the modified Ashworth scale and to zero or less on the Physician Global Impression of Change scale, or until the subject requested retreatment [194]. Dual criteria such as these are more difficult to meet and prolong the recorded duration.

Overall, the many different assessment methods for evaluating efficacy and duration of BoNT clinical effects necessitate careful inspection of the criteria used to define response. The different assessment methods further contribute to the difficulty comparing duration across studies. The statistically driven Kaplan–Meier analysis provides different insights into clinical trial outcomes than, for instance, time to retreatment, which itself provides different insights than return to baseline on a clinical rating scale. The assessments may be useful for different purposes but care must be taken not to directly compare data derived from such different methods.

#### 7.3.5. Rating Scales and Raters

The rating scale utilized is another study-level variable that can affect duration results. An example is from the glabellar lines literature, in which most BoNT products have been assessed on a 4-point categorical rating scale refined from the original Facial Wrinkle Grading System published by Keen, Blitzer, Brin, and others in 1994 [195]. The current 4-point scales were developed for registration purposes and are accompanied by proprietary, validated photonumeric guides used to inform the facial wrinkle ratings. Given that each of these guides uses different photos to define their ratings, they are not identical, making it difficult to draw conclusions about BoNT comparability across studies. In the therapeutic literature, different scales have been used to evaluate cervical dystonia (e.g., Cervical Dystonia Severity Scale [183], Toronto Western Spasmodic Torticollis Scale [187]) and spasticity (e.g., Ashworth scale [196], modified Ashworth scale [190], modified Tardieu scale [197], individual goal attainment scales [198]).

Across aesthetic and therapeutic uses, ratings may be performed by investigators who administer the injections, independent raters, and/or by the subjects themselves. The latter are included based on a growing recognition of the importance of patient perception of treatment effects and the lack of complete concurrence between patients and clinicians. Some studies measure duration based on the ratings of either investigators or subjects, whereas others use a composite of both investigator and subject ratings [199]. If duration is estimated based on loss of response on only one rating scale, it is likely to be shorter than if duration is estimated based on loss of response on two ratings scales or across 2 raters (i.e., composite endpoint), due to the lack of complete concurrence in ratings. Again, this complicates comparison of duration across studies.

#### 7.3.6. Follow-Up Timepoints

Follow-up timepoints also differ in BoNT studies, with variations in both number of timepoints assessed, time between assessments, and the duration of follow up. Studies designed with numerous follow-up timepoints enhance the precision of the comparison, regardless of the assessment method used [5].

### 7.4. Summary of Study-Level Variables

Comparing efficacy and duration from different studies, particularly if they used different rating scales and assessments, can be misinterpreted as one product having higher efficacy or a longer duration than another. Study-level variables can affect conclusions not only about efficacy and duration, but also about onset, adverse events, and other outcomes. These challenges are likely to intensify as more BoNT products enter the market.

Prospective studies designed to directly compare BoNT products are preferable to comparing across different studies because, in the former, the interventions are subject to the same protocol, tested in the same subject populations, and evaluated using the same outcome measures. However, prospective comparison studies have challenges of their own. In such studies, it can be difficult to select comparable doses of BoNT products given that units are not interchangeable and BoNT onset and duration vary with dose. Treatment paradigms, including number and location of muscles injected, vary between BoNT products, making it difficult to design studies with protocols optimized for each BoNT product. As noted above, these studies often have highly structured protocols that do not reflect clinical practice.

Although randomized, double-blind trials have the advantage of minimizing the effects of pre-existing differences and expectation on outcomes, designs can differ based on the types of conclusions that the authors seek to draw, such as non-inferiority or superiority studies. Non-inferiority trials are designed to show that the effect of one treatment is not inferior to that of an active comparator treatment by more than a specified statistical margin [200]. Conclusions about equivalence, or equipotency, cannot be made based on non-inferiority clinical trials not designed for that purpose, although such claims have erroneously been made in the BoNT literature [137,201,202].

Overall, study-level variables can influence conclusions about the comparability of BoNTs that may or may not be due to intrinsic product-level differences. It can be difficult to determine the source of the differences observed and it is important for clinicians to be aware that such ambiguity exists. Table 5 lists study-level variables to consider in comparing efficacy and duration of different BoNTs.

## 8. BoNT Safety and Adverse Events

The approved BoNT products are generally well-tolerated at approved doses. Adverse events differ based on indication and may be due to relaxation of the treated target muscle, local diffusion away from the injection area, or spread distant from the site of injection. Diffusion of BoNT products is based on intrinsic, product-level characteristics and has been discussed in the literature; readers are referred to several reviews [5,203]. Product-level local and distant diffusion characteristics influence the safety margin in preclinical models and adverse event profile in clinical situations [5].

In addition, in clinical practice, safety is linked to the dose administered. Unit potency, also an intrinsic BoNT product factor, is therefore critical to product safety: Dose confusion among products can have serious consequences for patients such that doses that are too low will not produce optimal efficacy and doses that are greater than desired can increase the risk of adverse events.

As described for efficacy and duration, extrinsic study-level factors can influence adverse event rates for BoNT products. An example of study-level factors affecting adverse event rates in clinical trials can be found in the cervical dystonia literature, where patients with pre-existing dysphagia are sometimes included or excluded from studies. Given that pre-existing dysphagia has been found to persist after BoNT treatment [204], excluding such patients can lead to a lower rate of post-treatment dysphagia obtained in the study. Another example comes from the neurogenic detrusor overactivity literature. A proportion of patients with this condition require clean intermittent catheterization to completely drain urine from the bladder and help prevent infection. Studies that include only patients who require catheterization at baseline do not count the need for catheterization as an adverse event [205]. In contrast, studies that include patients who do not catheterize at baseline report new catheterization as an adverse event [206,207]. These observations illustrate that, when comparing across clinical studies, it is important to consider the inclusion criteria of the study population and whether this may have influenced the adverse event rates obtained.

## 9. Immunogenicity

All foreign proteins injected into the body have the potential to induce the development of antibodies. In the case of BoNTs, the development of antibodies that interfere with clinical response is an uncommon occurrence [208,209,210,211,212,213], likely due to the extremely high potency and low amounts of protein injected. It will nevertheless be important to monitor the immunogenicity of newer BoNT products.

Antibodies that develop in response to protein therapies such as BoNTs are classified as neutralizing—meaning that they interfere with the drug’s action—and non-neutralizing—meaning that they do not interfere with the drug’s action. In some cases, antibodies that develop against the BoNT protein can be neutralizing, whereas antibodies that develop against the NAPs are non-neutralizing [214]. Some tests assess both neutralizing and non-neutralizing antibodies (e.g., the enzyme-linked immunosorbent assay [ELISA]), whereas in vivo tests such as the mouse protection assay assess only neutralizing antibodies [214,215].

### 9.1. Factors Affecting Neutralizing Antibody Formation

Over the years, numerous studies have evaluated factors that affect neutralizing antibody formation with the three established type A products (onabotA, abobotA, incobotA) and these have been reviewed [208]. Past studies found that the incidence of neutralizing antibody formation increased with the cumulative dose and number/frequency of injection visits [216,217]. However, in more recent studies, few subjects developed neutralizing antibodies regardless of number of treatment cycles or indication, suggesting that current treatment practices—which have been informed by the aforementioned past studies—contribute to the current low rates [208,209,210,211,212,213].

A limited number of studies have reported that switching to different BoNT/A products is a risk factor for neutralizing antibody formation [218,219]. Another study reported that switching secondary non-responders from one BoNT/A preparation to another reinstituted at least a partial response [220]. Given the extremely low rates of neutralizing antibody formation with the established BoNT/A products and the variability in responses even in patients who have developed neutralizing antibodies, potential reasons for these partial responses are that the doses and muscles involved were re-evaluated and updated or that patients’ titers varied over time, resulting in clinical non-response after one series of injections and clinical response after another.

### 9.2. Secondary Non-Response: Usually Not Due to Neutralizing Antibodies

The term secondary nonresponse has been used to describe inadequate or non-response to a pharmacological intervention following previously successful treatment. Secondary non-response can refer to a lack of pharmacological response to the medication or a suboptimal clinical response for other reasons. However, lack of perceived response to BoNTs is not usually due to a lack of pharmacological effect resulting from antibodies [212,213,221]. Instead, it is typically caused by inadequate dose, inappropriate muscle selection, complex movement patterns, and/or dynamic disease changes (e.g., contractures, abnormal postures, change in pattern of muscle contractions) [212,221,222]. A small study of cervical dystonia patients found that nonoptimal doses and muscles injected were the top two causes of secondary non-response [221]. Thus, an important first step in managing secondary non-response to BoNTs is to re-assess the doses and muscles injected.

Patient perceptions may also be a factor in secondary nonresponse. Patients sometimes interpret partial response as nonresponse [221]. Such patients may experience a therapeutic response but do not believe they are responding due to expectations. Patients may not recall how severe their condition was at baseline. After the first treatment, improvements from baseline are typically very evident to patients but improvements between the subsequent treatments may not be as noticeable because the change is not the same magnitude, as they did not return to baseline between treatments. Although secondary non-response to BoNTs can also be caused by the development of neutralizing antibodies, the vast majority of patients do not have them [213,221,223].

Additionally, the presence of neutralizing antibodies does not always lead to non-response. Studies have repeatedly shown that patients with neutralizing antibodies frequently show continued clinical response to BoNTs [213,218,224,225]. A study that followed 2240 patients who received up to 16 treatments with onabotA found that 11/2240 (0.49%) converted from antibody negative at baseline to positive at one or more post-treatment time points, but only three were clinically non-responsive at some point following a positive neutralizing antibody test [224]. This study also showed that neutralizing antibody status can vary over time, as only 4/2240 had a positive test at the final post-treatment study visit. A change in antibody status over time has also been reported by others [213,215,226] and, notably, in a recent meta-analysis of 5876 subjects treated with onabotA across 10 therapeutic and aesthetic indications in which 0.5% developed neutralizing antibodies at some point but only 0.3% remained positive at the end of the study [213].

### 9.3. Clinical Implications of Neutralizing Antibody Formation?

There is an imperfect relationship between neutralizing antibodies and clinical non-response [210,213,224,225]. Based on manufacturer’s sponsored clinical trials, doses, and injection intervals, the rates of neutralizing antibody formation with the established BoNT/A products are low: reported as 0% with onabotA in lateral canthal lines [3] and 1.2% in cervical dystonia [4]; 0% with abobotA in glabellar lines and less than 3% in cervical dystonia [7]; 0% with incobotA in glabellar lines and 1.8% in cervical dystonia [9]; 0% with daxibotA in glabellar lines and 0.5% in cervical dystonia [12]; and 0.14% with prabotA in glabellar lines [11]. The rate of neutralizing antibody formation with rimabotB is reported as 18% or less of cervical dystonia patients in the registration studies [13].

Several recent studies that have compared rates of neutralizing antibody formation across different BoNT products are retrospective chart reviews that were not designed for direct comparison. For example, different BoNT products were administered for different durations in at least one study [227], whereas others had large differences in patient numbers per group [218,219]. Moreover, doses were not controlled in these retrospective studies. These limitations preclude conclusions about relative immunogenicity among the different BoNTs.

Overall, the rates of neutralizing antibody formation with the established BoNT/A products are low with current labeled treatment recommendations.

## 10. Summary

An increasing number of BoNTs are currently available and in clinical development, some of which include innovations in serotype and formulation. BoNT products are not interchangeable due to differences introduced at each step of the manufacturing process: bacterial strain, fermentation, purification, excipients, finishing, and unit potency testing, all of which affect clinical profile. Of paramount importance for clinicians, units of BoNT products are not interchangeable due to differences in the assays used to measure unit potency, including different potency reference standards. Each BoNT has its own dosing information based on clinical studies in each indication; there are no established fixed inter-product dose ratios. Understanding the unique features of each BoNT is essential to optimizing patient experience, including efficacy, safety, and patient satisfaction.

**Figure 7:**
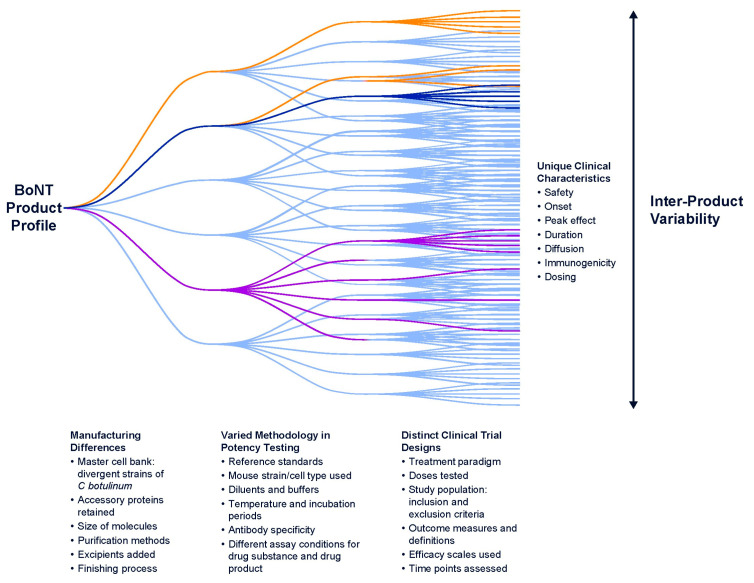
Lack of interchangeability among BoNT products. This graphic illustrates the divergence of BoNT products from manufacturing, through unit potency testing, to clinical trial designs. Individual lines illustrate the multiple points of divergence, with the highlighted orange, purple, and blue lines exemplifying different paths that products may take throughout the process. The intrinsic, product-level differences observed in the manufacturing and unit potency testing stages are compounded by study-level differences in clinical trial designs that ultimately lead to a unique set of clinical characteristics for each BoNT product.

In addition to the aforementioned intrinsic product-level differences between BoNTs, study-level differences contribute to the variability among products. Study outcomes such as efficacy and duration depend critically on the assessments and definitions of response. Moreover, all BoNT products exhibit dose responses—an observation that must be considered when comparing clinical properties such as duration. These study-level differences compound the intrinsic product-level differences, leading to unique clinical characteristics for each BoNT (Figure 7).

Non-interchangeability of BoNT products is recognized by regulatory agencies in major markets worldwide, which require a statement in the labels of all approved BoNT products indicating that units are not interchangeable or convertible among different BoNT products. To reinforce the individual potencies of BoNT products and prevent medication errors, the US Food and Drug Administration (FDA) requires each BoNT product to have its own unique nonproprietary name [228,229].

Given the non-interchangeability of BoNTs, issues related to non-medical switching among products take on added importance. As new BoNT manufacturers enter the market and existing manufacturers continue to negotiate pricing with institutions, governments and insurers, patients may be increasingly compelled to switch to products that are less expensive. This non-medical switching can disrupt the benefits of ongoing treatment, requiring clinicians to alter doses and injection sites to re-establish stable regimens for each patient, particularly because there are no fixed inter-product dose ratios. Such switching can also increase the potential for medical errors, adverse events, and cessation of treatment [230].

Overall, the growing number of BoNT products available or in development make this an intriguing time for BoNT therapy. This also includes emerging BoNT products with formulations designed for different onset of action and/or duration. The unique properties across this category of therapeutics highlight the importance for clinicians to recognize that each BoNT must be used according to its own specifications as supported by clinical studies, which will help decrease the potential for unexpected adverse events and maximize efficacy, duration, and patient satisfaction. With these considerations in mind, BoNT therapy has an exciting future of helping an increasing number of individuals achieve their treatment goals.

## Figures and Tables

**Table 2:** Ratios of doses studied or derived from onabotA:abobotA in clinical studies. Adapted from Ferrari et al., 2018 [114].

Indication	Author/Publication	RatioI (ona-botA:abobotA)
Blepharospasm	Bentivoglio et al., 2012 [115]	1:1.2–1:13.3
	Bihari, 2005 [116]	1:4–1:5
	Dodel et al., 1997 [117]	1:4–1:6
	Kollewe et al., 2015 [118]	1:2.3
	Marion et al., 1995 [119]	1:3
	Marchetti et al., 2005 [120]	1:3–1:11
	Nussgens and Roggenkämper, 1997 [121]	1:4
	Sampaio et al., 1997 [122]	1:4
Cervical dystonia	Bihari, 2005 [116]	1:4–1:5
	Dodel et al., 1997 [117]	1:4–1.6
	Marchetti et al., 2005 [120]	1:3–1:11
	Misra et al., 2012 [123]	3.1:1
	Odergren et al., 1998 [124]	1:3
	Ranoux D et al., 2002 [125]	1:3–1:4
	Rystedt A et al., 2015 [126]	1.7:1
	Van den Bergh and Lison, 1998 [127]	1:2.5
	Yun et al., 2015 [128]	1:2.5
Hemifacial spasm	Bihari, 2005 [116]	1:4–1:5
	Dodel et al., 1997 [117]	1:4–1:6
	Marion et al., 1995 [119]	1:3
	Van den Bergh and Lison, 1998 [127]	1:2.5
Spasticity	Bhakta et al., 1996 [129]	1:4–1:5
	Keren-Capelovitch et al., 2010 [130]	1:2.5
	Rasmussen et al., 2000 [131]	1:4

**Table 3:** Ratios of doses studied or derived from onabotA:incobotA in clinical studies.

Indication	Author/Publication	Ratio (onabotA:incobotA)
Blepharospasm	Bladen et al., 2020 [132]	1:1
	Juarez et al., 2011 [133]	1:1.2
	Kent et al., 2021 [134]	1:1.37
	Kollewe et al., 2015 [118]	1:1.2
	Roggenkämper et al., 2006 [135]	1:1
	Saad and Gourdeau, 2014 [136]	1:1
Cervical dystonia	Benecke et al., 2005 [137]	1:1
	Dressler et al., 2014 [138]	1:1
	Juarez et al., 2011 [133]	1:1.2
	Kent et al., 2021 [134]	1:1.21
Hemifacial spasm	Bladen et al., 2020 [132]	1:1
	Juarez et al., 2011 [133]	1:1.1
Spasticity	Italian Society of Pharmacology, 2017 [139]; Ferrari et al., 2018 [114]	1:1.5–1:2.5
Glabellar lines	Banegas et al., 2013 [140]	1:1
Blepharospasm	Kollewe et al., 2015 [118]	1:2

**Table 4:** Factors that affect BoNT efficacy and duration.

Intrinsic Product Factors
Serotype/subtype
Formulation
Unit potency
**Peripheral neuron type**
Motor (alpha and gamma)
Autonomic (sympathetic and parasympathetic)
Nociceptor
**Study-level factors**
Patient population
Dose
Injection paradigm
Efficacy and duration assessments
Rating scales/raters
Follow-up timepoint frequency

**Table 5:** Study-level variables to consider in comparing efficacy and duration of different BoNTs.

Patient population	Are there differences in clinical presentation, severity, or duration of disease, or of pre-existing conditions/comorbidities? Is a more or less responsive group included or excluded? Disease severity/complexity may influence overall efficacy, which can influence efficacy and duration.
Doses	Does the study account for dose–response effects in comparing products, in addition to non-interchangeability of units when evaluating efficacy and duration? Higher doses may lead to increased efficacy and longer durations.
Injection paradigm	Are the muscles and injection sites optimized for each of the BoNT products?
Efficacy and duration assessments	Are the definitions of efficacy and duration the same/comparable for the different products? For example, a definition that requires two raters to agree on an outcome is more difficult to achieve and will lead to longer duration than that of a single rater.
Rating scales/raters	Are the same rating scales being used? Who is doing the rating (e.g., investigator, subject)? Different scales and raters may yield different apparent responder rates and durations. Patient perception is an important outcome.
Follow-up timepoints	Is the number of timepoints adequate to provide a full assessment of duration? More follow-up timepoints give a more precise estimate of duration.

## Data Availability

This review was based on publicly available sources as cited in the reference list. No new data were created as part of this review.
